# 

**Published:** 1881-04

**Authors:** 


### CONTENTS:

ART. I.—Proceedings of Maryland and District of Columbia Dental Association. Annual Meeting.—(Continued.)......529
ART. II.—The Philadelphia Bogus Diplomas......543
ART. III.—Address to the Graduating Class of the Balto. College Dental Surgery. By Rev. Wm. Kirkus, M. A., LL. B.—(Continued.)......556
ART. IV.—Difficult Dentition. By Edward N Buckingham, M D......563

### EDITORIAL, ETC.

Advances and Retrogression in Medical Teaching.—Bellevue Hospital Medical College......572

### BIBLIOGRAPHICAL.

Elementary Treatise on Practical Chemistry and Qualitative Inorganic Analysis. By Frank Clowes......575
Essentials of Chemistry. Organic and Inorganic. By R. A. Witthaus, A M., M. D......576

### MONTHLY SUMMARY.

Bleaching Gutta Percha......576

